# Effect of low-level laser therapy on pain, quality of life and sleep in patients with fibromyalgia: study protocol for a double-blinded randomized controlled trial

**DOI:** 10.1186/1745-6215-13-221

**Published:** 2012-11-21

**Authors:** Paulo de Tarso Camillo de Carvalho, Ernesto Cesar Pinto Leal-Junior, Ana Carolina Araruna Alves, Caroline Sobral de Melo Rambo, Luciana Maria Malosa Sampaio, Claudia Santos Oliveira, Regiane Albertini, Luis Vicente Franco de Oliveira

**Affiliations:** 1 Post Graduate Program in Rehabilitation Sciences, Nove de Julho University (UNINOVE), Rua Vergueiro, 235, São Paulo, SP, 01504-001, Brazil; 2 Post Graduate Program in Biophotonics Applied to Health Sciences, Nove de Julho University (UNINOVE), Rua Vergueiro, 235, São Paulo, SP, 01504-001, Brazil

## Abstract

**Background:**

Low-level laser therapy (LLLT) has been widely used as adjuvant strategy for treatment of musculoskeletal disorders. The light-tissue interaction (photobiostimulation) promotes analgesic and anti-inflammatory effects and improves tissue healing, which could justify the recommendation of this therapy for patients with fibromyalgia, leading to an improvement in pain and possibly minimizing social impact related to this disease. The present study proposes to evaluate the effect of LLLT on tender points in patients with fibromyalgia, correlating this outcome with quality of life and sleep.

**Methods/design:**

One hundred and twenty patients with fibromyalgia will be treated at the Integrated Health Center and the Sleep Laboratory of the Post Graduate Program in Rehabilitation Sciences of the Nove de Julho University located in the city of Sao Paulo, Brazil. After fulfilling the eligibility criteria, a clinical evaluation and assessments of pain and sleep quality will be carried out and self-administered quality of life questionnaires will be applied. The 120 volunteers will be randomly allocated to an intervention group (LLLT, n = 60) or control group (CLLLT, n = 60). Patients from both groups will be treated three times per week for four weeks, totaling twelve sessions. However, only the LLLT group will receive an energy dose of 6 J per tender point. A standardized 50-minute exercise program will be performed after the laser application. The patients will be evaluated regarding the primary outcome (pain) using the following instruments: visual analog scale, McGill Pain Questionnaire and pressure algometry. The secondary outcome (quality of life and sleep) will be assessed with the following instruments: Medical Outcomes Study 36-item Short-Form Health Survey, Fibromyalgia Impact Questionnaire, Berlin Questionnaire, Epworth Sleepiness Scale and polysomnography. ANOVA test with repeated measurements for the time factor will be performed to test between-groups differences (followed by the Tukey-Kramer post hoc test), and a paired *t* test will be performed to test within-group differences. The level of significance for the statistical analysis will be set at 5% (*P* ≤0.05).

**Trial registration:**

The protocol for this study is registered with the Brazilian Registry of Clinical Trials – ReBEC (RBR-42gkzt)

## Background

According to the American College of Rheumatology (ACR), fibromyalgia is a chronic musculoskeletal condition characterized by generalized pain for at least three months in combination with sensitivity in 11 or more of the 18 points sensitive to palpation in different parts of the body, known as tender points [1,2]. Fibromyalgia was not a clinically well-defined condition before the 1970s, and unlike what was thought in the past, this is not an inflammatory condition and does not lead to joint impairment or deformities. However, the chronic nature of this condition has a negative impact on quality of life [3].

The most widely accepted theory is that fibromyalgia is caused by an abnormality in pain modulation mechanisms - a central nervous system disorder in regulating sensitivity to pain. Thus, an individual may exhibit a reduction in serotonin (neurotransmitter of the inhibitory descending system) and in increase substance P (neuroexcitatory substance involved in the conduction of pain) in the central nervous system [4].

Fibromyalgia is often associated with other syndromes, such as fatigue, sleep disorders, morning stiffness and psychological disorders, such as anxiety and depression. Sleep disorders are closely linked to somatic symptoms in patients with fibromyalgia and not to their personality. The most common complaints are difficulty falling asleep, frequent awakening during the night, difficulty getting back to sleep, restless superficial sleep and early rising, the consequences of which are nonrestorative sleep and a sensation of weariness, which contribute toward a poor quality of life. Thus, non-restorative sleep is an important aspect among the manifestations of fibromyalgia, which makes the investigation into primary sleep disorders essential to the assessment of affected individuals [5,6]. The complexity of the factors involved regarding both diagnosis and adequate treatment underscores the need for further studies aimed at broadening knowledge on the effects of this condition on patients.

There are several treatment options for fibromyalgia, such as drugs [7,8], psychotherapy [9], hydrotherapy [10], electrotherapy [11], exercise [12] and laser therapy [13,14]. However, there is no consensus or defined protocol regarding the use of these treatment options. Low-level laser therapy (LLLT) is one of the more recent pain treatment modalities in the field of physical therapy. According to Laakso *et al*. [15], the analgesic effect of phototherapy may be mediated by hormonal/opioid mechanisms, the responses of which depend directly on the dose and wavelength used to irradiate the tissue. Thus, the parameters directly affect the results. LLLT is also reported to act on peripheral neural stimulation and the regulation of microcirculation, interrupting pain mechanisms and promoting analgesia [16]. The normalization of microcirculation and the capacity of neural transmission obtained through laser therapy have been described as responsible for the interruption of the vicious circle that gives rises to and perpetuates pain [13].

In last ten years, important advances have occurred in the therapeutic use of photobiostimulation with low-level lasers. Such advances have occurred due to a greater understanding of the mechanisms of action involved as well as technological developments and the publication of relevant studies and research related to the advantages obtained with the use photobiostimulation in clinical practice. However, the lack of consensus regarding therapeutic protocols hinders multicenter comparisons of the many clinical trials published. Thus, a number of scientific papers have been drafted to establish a therapeutic protocol for the use of photobiostimulation with regard to exposure time, intensity, energy density, application mode and application site.

A considerable number of studies have demonstrated the remission of pain in patients with fibromyalgia through the use of LLLT. However, this is not yet a well-established therapy and comparisons of controlled clinical trials are rare. Moreover, there are problems regarding the standardization of treatment [13,14,17,18]. In the investigation of complex phenomena such as fibromyalgia, the application of multiple methodological techniques enables a better assessment and understanding of such phenomena [19].

The aim of the proposed study is to assess the analgesic effect of LLLT on tender points in patients with fibromyalgia and establish correlations with quality of life and sleep.

## Methods and study design

### Characterization of proposed study

**Figure 1:**
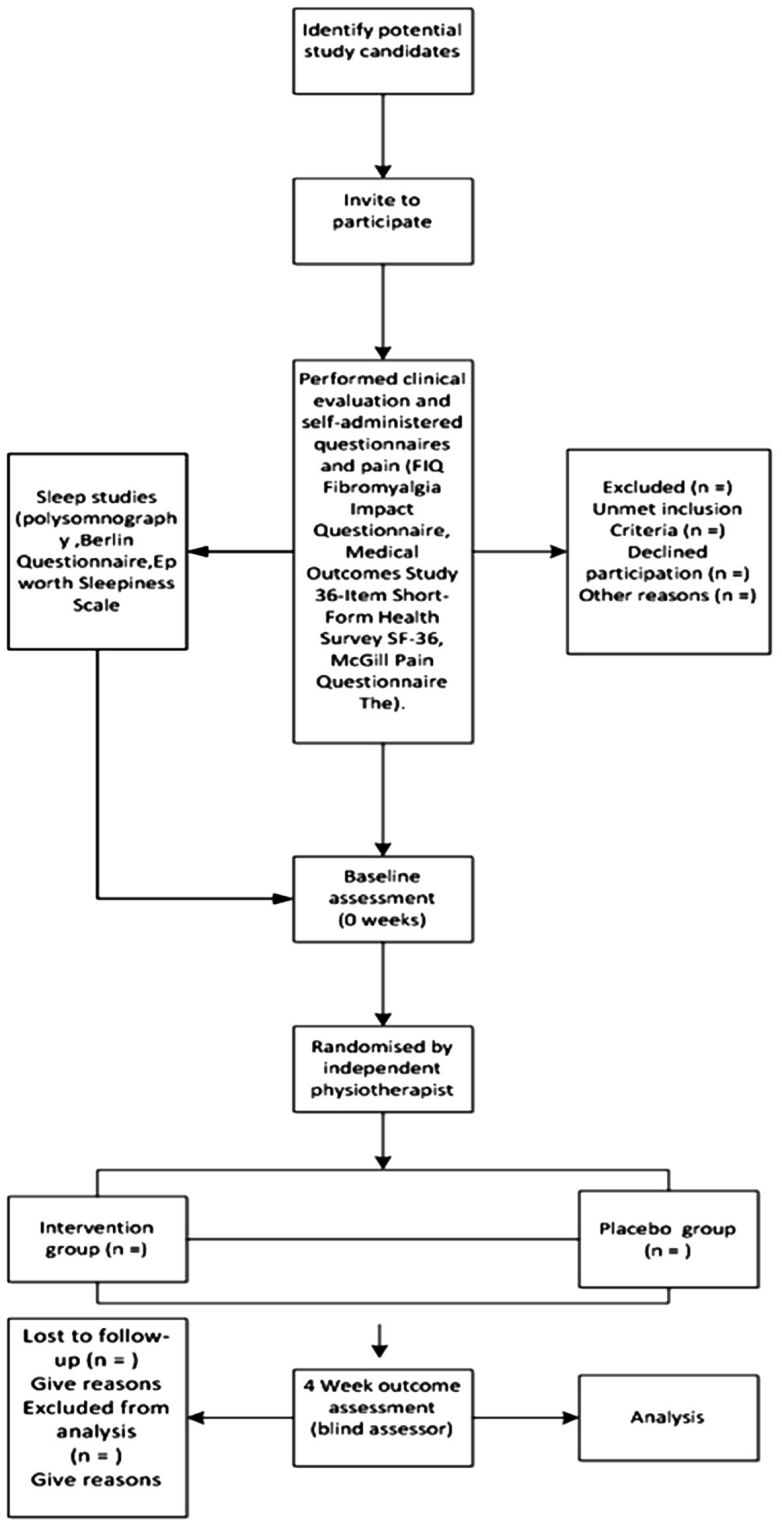
**Flow chart characterization of the experimental design,sample composition and trial protocol.**

A double-blind randomized controlled trial will be carried out at the Sleep Study Laboratory of the Post Graduate Program in Rehabilitation Sciences of the Nove de Julho University (UNINOVE) and the UNINOVE Integrated Health Center, located in the city of Sao Paulo, Brazil. (Figure 1)

### Characterization of subjects

The sample will be comprised of 120 patients with fibromyalgia with a clinical diagnosis based on the ACR criteria. The subjects will be screened at the UNINOVE Rheumatology Clinic.

### Composition of sample

The required sample size was determined for the primary outcome variable, that is, overall score of the Fibromyalgia Impact Questionnaire (FIQ). According to Bennett *et al*. [20], a clinically relevant change is a 15 to 20% reduction in the total FIQ score (which is equal to an approximate 10 to 15 points reduction). Assuming a unilateral alternative (that is, the intervention reduces the impact of fibromyalgia), we can detect differences of at least 15% with a power of 95% and α of 0.05 with two groups (intervention and usual control group) of 45 participants, with a mean in the FIQ of approximately 70 and a standard deviation of approximately 20 points. Assuming a maximum loss of follow-up of 30%, we will recruit a total of 60 women with fibromyalgia for each group. We will recruit a total of 120 women with fibromyalgia (that is, one LLLT intervention group and control group of 60 persons each).

### Inclusion criteria

The following criteria will be adopted as requirements for inclusion in the study:

•A clinical diagnosis of fibromyalgia based on ACR criteria;

•Cognitive level sufficient for understanding the procedures and following the instructions;

•Agreement to participate in the study and the signing of a statement of informed consent after receiving clarifications regarding the objectives of the study.

### Exclusion criteria

Patients with the following will be excluded from the study:

•Psychiatric disorders, history of drug abuse or other behavior that requires psychiatric medication intervention;

•Development of situations beyond clinical control that do not allow participation in aerobic training activities, exertion or group activities;

•Severe physical disability;

•Co-morbid condition (irritable bowel syndrome, intestinal cystitis, and others);

•Uncontrolled endocrine disorders;

•Although there is no description of serious complications in individuals submitted to LLLT, those with a history of epilepsy, seizures, heart disease, arrhythmia, pacemaker, undiagnosed pain, pregnancy and malignant tumors will be excluded from the study.

### Composition of groups and randomization

After the initial evaluation, the 120 volunteers will be randomly allocated to an intervention group (LLLT; n = 60) or control group (CLLLT; n = 60). Randomization numbers will be created using a randomization table at a central office, where a series of sealed, opaque, numbered envelopes will be used to ensure confidentiality. Each envelope will contain a card indicating the group in which the patient will participate. After fulfilling the eligibility criteria, a clinical evaluation and assessments of pain and sleep quality will be carried out and self-administered quality of life questionnaires will be applied (Fibromyalgia Impact Questionnaire, Medical Outcomes Study 36-item Short-Form Health Survey and McGill Pain Questionnaire). After the volunteer signs the statement of informed consent, she will randomly select one of the envelopes. This study received approval from the UNINOVE Human Research Ethics Committee (protocol number 474921). Additionally, the study is registered with the World Health Organization Universal Trial Number (UTN) U1111-1125-5007 and the Brazilian Registry of Clinical Trials (number - RBR-42gkzt).

### Blinding procedures

A single examiner with the necessary skills will perform the evaluations in order to achieve reproducible examinations and more reliable results. A second examiner will be trained to administer the laser and conduct the exercises. A third examiner will perform the re-evaluations of the two groups. The groups will not be identified in order to avoid ‘unmasking’ during the study.

## Experimental protocol

### Evaluation and educative session

The evaluations will occur prior to and at the end of the therapeutic program by a researcher with no knowledge regarding the type of treatment to which the subjects will be submitted. All subjects in both groups will be submitted to the same evaluation protocol, which will include the aspects listed below.

#### Personal data

Age (in years), body mass (in kilograms), height (in meters), profession, schooling (no schooling, elementary education, high school education, university education, postgraduate education) and marital status.

#### History of pain

Duration of pain, sites of pain in decreasing order of intensity, period of day in which pain is more intense, factors of improvement and worsening of pain, sleep quality, type of mattress and pillow.

#### Visual analog scale for pain

A visual analog scale will be used for the assessment of pain intensity, consisting of a straight line measuring 10 centimeters, with no numbers displayed and on which the words ‘no pain’ appear on the left extremity and ‘unbearable pain’ appear on the right extremity. The patient will be instructed to mark a point on the line that indicates the intensity of pain she is feeling at the time. Higher scores denote greater pain intensity.

#### Pressure algometry of pain threshold

A pressure algometer (digital algometer, model DDK 20, manufactured by Kratos Ltd., Cotia, Sao Paulo, Brazil) is a device that assesses the pain threshold. This device consists of a round rubber disk (1 cm^2^) attached to a pressure (force) gauge. The gauge displays values in kilograms. Because the surface of the rubber tip is 1 cm^2^, the readings are expressed in kilogram per square centimeter (kg/cm^2^). The range of the algometer is 0 to 10 kg and records in 0.1 kg divisions. At one end of the device, perpendicular pressure is applied to the surface of the skin and recorded using a manometer. The algometry of the pain threshold on the tender points will be performed based on the fibromyalgia classification criteria of the ACR, following the procedures described below:

•The patient will be asked to sit in a chair with feet supported on the ground and hands supported on the lap;

•The tender points will be marked with a skin marking pen and evaluated bilaterally in the following order: occipital, anterior lower cervical, trapezius, supraspinatus, second costochondral joint, lateral epicondyle and medial edge of the knee;

•The patient will be instructed to remain in an orthostatic position and the tender points of the gluteus and greater trochanter will be marked with the pen, bilaterally;

•During the assessment of the pain threshold on the tender points, perpendicular pressure will be applied to the surface of the skin and increased gradually in increments of 0.1 kg until the patient reports feeling pain;

•Positive results will be those below 2.6 kg/cm^2^, which is equivalent to the 4.0 kg/cm^2^ described in studies by Wolfe *et al*. [21].

#### Fibromyalgia Impact Questionnaire (FIQ)

The version of the FIQ translated into Portuguese and validated by Marques (2006) will be used. The FIQ is a quality of life assessment tool specific to fibromyalgia. The questionnaire has items addressing functional capacity, professional situation, psychological disorders and symptoms, for which higher scores denote a greater impact from fibromyalgia on quality of life.

#### Medical Outcomes Study 36-item Short-Form Health Survey (SF-36)

The version of the SF-36 translated into Portuguese and validated by Ciconelli *et al*. [22] will be used. This is a generic quality of life assessment tool consisting of 36 items distributed among eight components: functional capacity (10 items), physical aspects (4 items), pain (2 items), general health state (5 items), vitality (4 items), social aspects (2 items), emotional aspects (3 items) and mental health (5 items) as well as 1 item soliciting a comparative assessment of current health status with health status one year earlier. Each component ranges from 0 to 100 points, with higher scores denoting a better quality of life.

#### McGill Pain Questionnaire

The McGill Pain Questionnaire (MPQ) adapted to the Portuguese language will be used [23]. The questionnaire consists of a list of 78 pain descriptors organized into 4 major categories (sensory, affective, evaluative, and miscellaneous) and 20 subcategories, each made up of at least 2 and up to 6 words, to which intensity values are assigned. To calculate Pain Rating Index (PRI) the descriptors of the third category, which characterizes pain, will be used. Present Pain Intensity (PPI) will be evaluated using a verbal scale of pain (0 - no pain, 10 - very strong pain).

As some patients may have difficulty reading and understanding the instructions of the questionnaires (McGill Pain Questionnaire, SF-36 and FIQ), the reading will be performed together with the researchers.

#### Sleep assessment

The sleep studies will be carried out at the Sleep Laboratory of the Post Graduate Program in Rehabilitation Sciences (UNINOVE) through complete nocturnal standard level I polysomnography using the Somnologica Studio (Embla A10 version 3.1.2, Medcare Flaga, Hs. Medical Devices, Reykjavik, Iceland), composed of 16 channels for the monitoring of electroencephalogram, electrooculogram, electromyography of the submentum and tibialis anterior muscles and electrocardiogram, including channels for digital oximetry, nasal pressure (canulla), pressure transducer, respiratory movements (Xtrace thoracic and abdominal straps) and body position. The assessments will be performed manually by a specialized reader following the guidelines of the American Academy of Sleep Medicine [24], and the criteria of the Brazilian Sleep Society and the report of the results will be drafted by a specialist in sleep medicine. The Berlin Questionnaire will be administered also. This 10-item patient history questionnaire has recognized efficacy in differentiating individuals with a greater risk of sleep apnea. Moreover, the Epworth Sleepiness Scale (Johns, 1991) [25] will be employed for the assessment of excessive daytime sleepiness.

Regarding the performance of the examinations and procedures during the sleep study, the patients will be instructed to remain as relaxed as possible and sleep naturally as if at home. All signals will be recorded continuously throughout the entire sleep period of each subject.

#### Educative session

The educative session will be individual and carried out following the initial evaluation. The subject will be informed with regard to the nature of fibromyalgia, symptoms, treatment (stressing the importance of exercise) and consequences for activities of daily living. Some exercise strategies will be taught, such as extra body weight loading and adequate positions for the performance of activities of daily living and practice (work). A basic exercise program will then be taught, composed of 11 exercises for stretching the gluteus, paravertebral, hamstrings, pectoral, scalene, intercostals and trapezius muscles. The patients will receive a chart with all the information on fibromyalgia as well as illustrations and descriptions of the exercises to perform. The chart will be furnished to all patients in order to increase compliance with the at-home exercise program.

### Low-level laser therapy

#### Specifications

The Twin Laser (MM Optics Ltda., São Carlos, São Paulo, Brazil) will be employed, the active medium of which is made up of a Gallium-Aluminum-Arsenide semiconductor diode, with emission in the 780 nm near infrared wavelength (invisible) and variable power values in the continuous emission mode. The display provides the doses according to the power and application time. The laser will undergo verification of functioning, energy source, points of application and measurement of power.

To provide the ‘blinding’ of the study, the laser equipment has two identical application points furnished by the manufacturer - one active and the other a placebo (that does not emit energy); both have a sound device and guide light. The points will be named A and B by a researcher who will not participate in the treatment or evaluations. The researcher performing the applications and the evaluators will be unaware of which points received the effective dose and which received the placebo. The patients will also not have access to information regarding to which group they belong. The points will be identified only at the end of the data acquisition phase.

#### Application of laser

Both groups will receive three laser applications per week for four weeks, totaling twelve sessions. The applications will be performed in spot form with direct contact between the laser probe and the skin. However, only the patients in the effective radiation group with receive an energy dose of 6 J per point. The application points will be performed at the tender points identified prior to each session.

### Exercises

Both groups (LLLT and control) will undergo a 50-mintue exercise program following the application of the laser or placebo, which will consist of 10 minutes of warm up and stretching, 30 minutes of aerobic exercise on a treadmill and 10 minutes of relaxation and stretching, three times a week throughout the four weeks of the study.

### Evaluation of results

The intention-to-treat analysis will be followed. The patients will be evaluated with regard to the primary outcome (pain) using the visual analog scale for pain, the McGill Pain Questionnaire and pressure algometry. The secondary outcome (quality of life) will be assessed using the SF-36 and FIQ. The use of medication will be controlled throughout the study. The data on the use of analgesics and daily dosage of adjuvant therapy, such as antidepressants and muscle relaxers, will be recorded and tabulated.

### Statistical analysis

ANOVA test with repeated measurements for the time factor will be performed to test between-groups differences (followed by the Tukey-Kramer post hoc test), and a paired *t* test will be performed to test within-group differences. The level of significance for the statistical analysis will be set at 5% (*P* ≤0.05).

## Discussion

According to data obtained from World Health Organization, a large number of people around the world suffer with chronic pain and it reflects in physical incapacity related to work activities. Furthermore, chronic pain represents a limiting factor in quality of life in modern society. Recent studies have shown that 10% of world population suffers with diffuse chronic pain, and 2 to 5% of these cases are related to fibromyalgia [26-28].

Billions of American dollars are expended around the world every year in diagnostics, treatment, lost workdays and judicial process due chronic pain. In some cases, the cause of pain can be due fibromyalgia. Therefore, the development of efficient therapies can also represent an important decrease in financial costs in relation to chronic pain related to musculoskeletal disorders [29-31].

LLLT has been widely used in treatment of skeletal muscle disorders with positive results [32-35]. The light-tissue interaction (photobiostimulation) leads to analgesic and anti-inflammatory effects [36], and also improves tissue healing [37]. With this perspective in mind, we can expect that LLLT in addition to exercise will significantly decrease pain and improve quality of life in these patients.

Finally, with the development of the protocol of this study, it is expected that the group treated with LLLT will present with significant reduction of pain intensity on the visual analog scale, increase in the pressure pain threshold, and improvement in the overall quality of life and quality of sleep.

## Trial status

At the time of manuscript submission the patients are being recruited to trial.

## Competing interests

The authors declare that they have no competing interests.

## Authors’ contributions

All the authors contributed to the conception and design of the study. LVFO, ACA and PTC provided the idea for the study, established the hypothesis and wrote the original proposal. ECPLJ and PTC significantly contributed to writing this paper, while CSMR,RA, LMMS and CSO were involved in critically revising the manuscript. PTC and ACA wrote this protocol paper with input from all co-authors. All authors read and approved the final manuscript.
